# Abiotic Stress Reshapes Rhizosphere Community Assembly and Tea Quality: Root Exudates, Plant–Soil Interactions and Microbial Management

**DOI:** 10.3390/plants15121869

**Published:** 2026-06-16

**Authors:** Yujie Song, Hiroto Yamashita, Takashi Ikka

**Affiliations:** 1 The United Graduate School of Agricultural Science, Gifu University, 1-1 Yanagido, Gifu 501-1193, Japan; song.yujie.x7@s.gifu-u.ac.jp (Y.S.); yamashita.hiroto@shizuoka.ac.jp (H.Y.); 2 Faculty of Agriculture, Shizuoka University, 836 Ohya, Shizuoka 422-8529, Japan; 3 Institute for Tea Science, Shizuoka University, 836 Ohya, Shizuoka 422-8529, Japan; 4 Research Institute of Green Science and Technology, Shizuoka University, 836 Ohya, Shizuoka 422-8529, Japan

**Keywords:** *Camellia sinensis*, abiotic stress, rhizosphere microbiology, root exudates, functional microorganisms, tea quality

## Abstract

Abiotic stresses affect the growth of tea plants (*Camellia sinensis*) and reduce their yield and quality. The tea plant is a perennial crop. Its adaptability to abiotic stresses and the formation of quality depend not only on internal physiological regulation, but also on long-term interactions with the surrounding soil environment. However, how abiotic stresses reshape the tea rhizosphere community structure, and the knowledge of how these changes shape tea quality remains limited. This review summarizes current knowledge on the tea rhizosphere microbiome under abiotic stress. First, we examine how stress reshapes microbial communities, including their composition, metabolic functions, interaction networks, and the recruitment driven by root exudates. Second, we explore the mechanism of rhizosphere microorganisms affecting tea plants, including participation in nutrient cycling, interaction mediated by exudates, and the regulation of secondary metabolic pathways related to the quality of tea. Finally, we discuss several nutrient-based and microbiome-based management strategies, such as the use of combined fertilizer, intercropping, PGPR, AMF, and SynComs. This review connects stress physiology, rhizosphere ecology, and tea quality regulation within a microbiome-centered framework, providing a basis for strategies that enhance stress tolerance and tea quality stability in the tea plant.

## 1. Introduction

As climate change is accelerating, the frequency and intensity of the abiotic stresses are rising, which have become key environmental factors that restrict the growth and development of crops and affect the quality formation [1,2]. Tea (*Camellia sinensis* (L.) O. Kuntze) is a perennial cash crop with a long growth cycle, continuous root activity, and high sensitivity to environmental changes. Abiotic stress directly influences the growth, development, and yield formation of tea plants. It can also affect tea quality by altering the accumulation of amino acids, catechins, and aromatic compounds in tea. These changes are associated with alterations in carbon and nitrogen metabolism, secondary metabolic pathways and signal regulation processes [3,4]. Therefore, balancing stress resilience with quality stability has become a central challenge for both tea cultivation and research.

Current research on abiotic stress in tea plants mainly focuses on the regulatory mechanisms at the molecular and physiological level. It primarily revolves around processes such as photosynthetic regulation, osmotic regulation, defense systems and metabolic regulation [5,6,7,8]. These studies have laid an important foundation for revealing the direct responses of tea plants to stress. However, it is difficult to systematically explain the ecological basis of their long-term adaptability and quality formation from plant physiology alone; it also depends on rhizosphere ecological processes and the plant–soil interaction [9,10].

The rhizosphere microbiome plays an active part in how plants cope with abiotic stress. Under stress, plants change both the amount and types of compounds released from their roots, with soluble sugars, amino acids, organic acids, and secondary metabolites typically secreted at higher levels [11,12,13]. These compounds provide carbon to rhizosphere microorganisms and act as chemical signals that influence microbial recruitment. The resulting changes extend to nutrient cycling, soil physicochemical properties, and the microbe-derived signals related to plant metabolism [11,14,15]. Through this exchange, rhizosphere microorganisms help tea plants maintain physiological stability under abiotic stress and shape the biosynthesis of quality-related metabolites, including amino acids, phenolics, and aroma precursors [16,17]. These processes can be summarized as a “stress-exudate-microbiome-quality” framework [18]. In this process, abiotic stress first changes root exudation composition, exudate-mediated recruitment reshapes the rhizosphere microbiome, and microbial functions ultimately influence the tea quality compounds accumulated.

Although increasing attention has been paid to abiotic stress responses, rhizosphere microorganisms, and tea quality formation, these topics are still often studied separately. As a result, the mechanistic links between stress-induced root exudates, microbial community assembly, rhizosphere functional metabolism, and the accumulation of quality-related metabolites in tea plants remain insufficiently integrated. Therefore, this review synthesizes current knowledge on tea plant–rhizosphere microbiome interactions under abiotic stress, with a particular focus on microbial community dynamics, root exudate-mediated recruitment, functional microbial metabolism, and quality regulation. We further discuss how this knowledge can be translated into microbiome-based management strategies for improving tea plant resilience and stabilizing tea quality under changing environmental conditions.

## 2. Major Abiotic Stresses Affecting Tea Plant Physiology and Quality

### 2.1. Drought Stress

Drought is one of the most widespread abiotic stresses affecting tea production, posing a persistent threat to tea plant growth and quality stability. Under drought, tea plants close their stomata and reduce transpiration to limit water loss. This also lowers CO_2_ uptake, slows photosynthesis, thereby reducing the supply of sugars and starch needed for energy metabolism in leaves [19]. Prolonged drought stress induces a series of protective responses in tea plants. These include ABA-mediated regulation, osmotic adjustment, and the accumulation of stress-related proteins and antioxidant enzymes that mitigate reactive oxygen species damage [20,21]. Drought stress also affects multiple metabolic pathways in tea plants, including photosynthesis, sulfur metabolism, and phenylpropanoid biosynthesis [7,8,22]. These changes may contribute to stress adaptation while simultaneously influencing tea quality-related metabolites.

Several studies have reported lower concentrations of catechins, caffeine, theanine, and other amino acids in tea leaves under drought stress [21,23]. Reduced accumulation of these compounds is often associated with decline in tea quality. Changes in aroma-related metabolites have also been observed under drought stress. For example, moderate drought can increase compounds associated with floral and fruity aromas, such as linalool, while decreasing certain fatty acid-derived volatiles [24,25]. Overall, drought stress affects both tea growth and quality formation. The associated physiological and metabolic adjustments may also change root exudation patterns, which could further influence rhizosphere microbial communities and their potential roles in tea quality regulation.

### 2.2. Salt Stress

With the intensification of soil salinization, salt stress has become one of the major abiotic stress factors limiting crop growth and quality formation. As an acidophilic cash crop, tea plants are particularly sensitive to salt stress. Through the combined effects of ionic toxicity and osmotic stress, salt stress disrupts physiological and metabolic processes in tea plants, thereby reducing tea yield and quality [26]. Under salt stress, tea plants close their stomata to reduce water loss, while Na^+^-induced damage to photosystem II further lowers photosynthetic efficiency. The excessive accumulation of reactive oxygen species also disrupts antioxidant homeostasis and contributes to cellular damage [27,28]. Salt stress also reshapes primary metabolism, especially carbon and nitrogen pathways, altering amino acid and sugar accumulation patterns in leaves [29,30].

Tea quality compounds are also affected by these physiology disturbances. Mild-to-moderate salt stress has been found to promote the accumulation of theanine and other free amino acids in tea plants. This response has been linked to the upregulation of theanine synthetase-related proteins, suggesting a promotive effect of salt stress on theanine biosynthesis in tea plants [31,32]. In contrast, severe stress is often accompanied by significant reductions in the contents of theanine, EGCG, and caffeine in tea leaves [28]. Overall, salt stress affects tea quality by disrupting plant growth and metabolic processes, while altered rhizosphere nutrient inputs and ionic conditions may further drive microbial functional reorganization.

### 2.3. Heat Stress

Unlike drought and salt stress, heat stress usually involves a rapid temperature increase, which disrupts cellular metabolism and reduces tea yield [33,34]. Heat stress frequently causes membrane injury and increases electrolyte leakage in tea leaves. It may also impair chloroplast integrity and photosystem II activity, reducing chlorophyll levels and photosynthesis capacity [34,35]. Heat stress also promotes the production of reactive oxygen species (ROS), such as hydrogen peroxide and superoxide anion. The excessive ROS accumulation may reduce antioxidant capacity while enhancing lipid peroxidation and protein damage [34].

Reduced accumulation of theanine in tea leaves under heat stress has been linked to altered expression of genes involved in theanine biosynthesis [36]. Since theanine is a major contributor to umami taste, decreases in theanine accumulation may negatively affect tea quality. Heat stress can additionally influence polyphenol metabolism through heat shock transcription factor activity, with subsequent effects on jasmonate signaling and reductions in flavonoid and catechin contents [37,38]. Previous studies have reported the involvement of auxin-related and gibberellin-related signaling pathways in regulating caffeine biosynthesis under heat stress. Under heat stress, elevated IAA and gibberellin levels are associated with the suppression of key genes in the purine degradation pathway, contributing to increased caffeine accumulation [39]. Auxin-producing rhizobacteria may also influence shoot IAA levels, suggesting a possible link between rhizosphere microbial activity and tea quality formation. In addition, auxin regulates root architecture by controlling the expression of biosynthesis genes and transporters, which affects the spatial pattern of root exudates and the potential for microbial colonization. Rhizobacterial IAA can supplement plant auxin levels, suggesting feedback between hormone status and rhizosphere microbial activity under stress [40]. Heat treatment can also alter the volatile composition of tea leaves, including compounds associated with floral and fruity aromas [38]. High temperature exposure induces substantial physiological and metabolic changes in tea plants, which may further influence rhizosphere microbial communities through alterations in root function and carbon allocation to the rhizosphere.

### 2.4. Cold Stress

Cold stress negatively affects cell membrane stability, photosynthetic, and metabolic activity in plants, with consequent reductions in crop yield and quality [41,42]. In tea plants, exposure to cold stress leads to a series of physiological and biochemical responses that may affect growth and quality formation [43]. Cold stress is frequently accompanied by reduced photosynthetic activity and enhanced membrane lipid peroxidation in tea leaves. Suppression of antioxidant enzyme activity may further intensify oxidative stress and inhibit plant growth [44]. Producing volatile organic compounds (VOCs) is considered an important response of tea plants to cold stress. These compounds have been linked to calcium-related signaling pathways, enhanced antioxidant enzyme activity and improved cold tolerance [45].

Multi-omics studies indicate significant metabolic changes in tea plants exposed to cold stress. Cold-responsive transcription factors have been linked to enhanced lignin biosynthesis under cold conditions. Accumulation of several metabolites, such as lipids, flavonoids, amino acids and catechin-related compounds, is also commonly observed, while glycoside levels may decrease [46,47]. Cold stress also affects volatile metabolites in tea leaves, especially floral-related and terpene-related compounds [48]. Low temperatures can affect both tea plant growth and metabolite accumulation through multiple physiological and metabolic responses. These effects may further influence the rhizosphere via changes in root carbon and nitrogen allocation as well as community structure.

### 2.5. Heavy Metal Stress

Heavy metal stress can negatively affect tea plant physiology. Studies have reported chloroplast damage, lower chlorophyll content, and reduced stomatal conductance in tea leaves exposed to excessive metal accumulation [49]. Tea plants often show reduced photosynthetic performance and weaker growth under heavy metal stress [50]. The excessive accumulation of ROS and increased membrane lipid peroxidation have also been reported, indicating enhanced oxidative stress in plant tissues [51]. In addition, high concentrations of heavy metals impair antioxidant defense systems by decreasing the activities of antioxidant enzymes, thereby further aggravating oxidative injury [50].

Heavy metal stress reshapes metabolic pathways involved in tea quality formation, inhibiting nitrogen metabolism and the accumulation of caffeine and free amino acids, while enhancing the carbon-based secondary metabolism, including organic acid, catechin, and lignin biosynthesis [49,52]. Heavy metal stress also reshapes polyphenol metabolism in a concentration-dependent manner. Moderate stress levels may stimulate the accumulation of tea polyphenols and proline to enhance antioxidant capacity and osmotic regulation, whereas excessive metal concentrations inhibit phenolic biosynthesis and impair metabolic balance [53]. The rhizosphere represents a key site for plant–soil–microbe interactions under heavy metal stress. Therefore, heavy metal stress can reshape rhizosphere microbial assembly by altering root metabolism, exudation profiles, and soil physicochemical conditions, thereby affecting microbial stress-mitigating functions.

## 3. Rhizosphere Microbiome Responses Under Abiotic Stress

Besides impacting tea plants, abiotic stresses also modify rhizosphere conditions. These changes reshape the microbial community structure and ecological functions at multiple levels, including community diversity, community composition, functional metabolism, and interaction networks.

### 3.1. Microbial Diversity and Community Assembly

Abiotic stresses change the rhizosphere microenvironment, thereby reshaping microbial community assembly. The effects of abiotic stress on microbial community diversity show different patterns, both across different diversity indices and stress types. For example, under drought stress, rhizosphere bacterial and fungal richness can remain relatively stable, although the relative abundance and evenness of communities often change [54,55,56]. In contrast, salt stress and heavy metal stress tend to cause stronger declines in alpha diversity of both bacterial and fungal communities [57,58]. At larger scales, rising global temperatures have been shown to reduce soil microbial diversity over the long term [59].

Abiotic stress is also a key environmental driver of directional microbial community assembly. Numerous studies have shown that temperature is a crucial driver shaping rhizosphere microbial communities. Distinct bacterial community compositions form under different temperature conditions, further influencing taxonomic structure and interspecies interaction networks [60,61]. Under salt or heavy metal stress, beta diversity analyses often show clear separation in microbial community structure between stress and control treatments, reflecting the strong reshaping effects of abiotic stress on microbial community assembly processes [58,62,63]. Although different abiotic stresses act through different mechanisms, they commonly reshape microbial community assembly through environmentally driven shifts in community composition.

Based on these findings, we summarized and compared the main enriched and depleted taxa in rhizosphere microbial communities under different abiotic stress conditions (Table 1). Because evidence regarding rhizosphere responses in tea plants remains limited under several abiotic stresses, representative studies from other plant systems were also incorporated to identify conserved microbial response patterns. Across these studies, microbial responses showed both commonalities at the taxonomic level and clear stress specificity. Among them, microbial groups with strong stress tolerance were repeatedly enriched under various stress conditions. At the phylum level, Firmicutes, Proteobacteria, Actinobacteria, and Bacteroidetes were frequently dominant, while at the genus level, *Sphingomonas* and *Bacillus* were commonly detected. This phenomenon further indicates that abiotic stress filters out environment-sensitive groups, while stress-tolerant groups become dominant and drive the restructuring of the microbial community. Given the species-specific nature of plant–microbe interactions and the functional differences among rhizosphere bacteria, endophytes, and AMF, these patterns should be viewed as provisional and updated as more tea-specific evidence emerges across cultivars, environments, and stress conditions.

### 3.2. Functional and Metabolic Responses of Microbial Communities

Under abiotic stress, soil microorganisms respond not only by changing community structure but also by remodeling their functional metabolism. The specific functional pathways that are reshaped depend on the type of stress, and these shifts often translate directly into changes in plant performance under adverse conditions. Niu et al. demonstrated that under drought stress, the functional composition of microorganisms is significantly reconstructed, with enrichment of metabolic pathways related to plant drought tolerance [84]. These metabolic functions concentrate on osmotic regulation, secondary metabolite synthesis, and plant hormone-related metabolism, which together support plant adaptation to drought stress. Xu et al. found that under salt stress, functions related to amino acid and lipid transport and metabolism, energy generation and transformation are significantly enriched in soil [69]. These functions can help maintain microbial osmotic regulation and energy homeostasis. They also support plant growth indirectly by enhancing defense mechanisms and signal transduction pathways under salt stress. Under heavy metal stress, Chen et al. reported that nitrogen functional metabolism in the soil’s microbial community is significantly reshaped [85]. Specifically, nitrifying and nitrite-oxidizing microorganisms are enriched, leading to elevated soil nitrate levels. This enhanced microbial nitrogen metabolism coincides with inhibition of root amino acid metabolism in plants, suggesting nitrogen competition between plants and microbes [85].

Therefore, different abiotic stresses can significantly influence adaptive processes of the plant-rhizosphere system by reshaping the functional metabolic profiles of soil microorganisms. These functional metabolic shifts can also change the nutrient allocation patterns between plants and microorganisms, intensifying resource competition for shared resources [86,87]. Ultimately, they feed forward into plant–microbe interactions that shape tea growth and the metabolic chemistry of tea quality.

### 3.3. Microbial Interaction Networks and Keystone Taxa

In addition to changes in community composition and function, abiotic stresses also reshape the structure of rhizosphere microbial interaction networks. Within these networks, keystone taxa often play key roles in shaping how the microbiome responds to stress. Under salt stress, network complexity increases with increasing salinity, with simultaneous increases in nodes, connection density, and modularity. A modular network structure with functional bacteria as the core is formed, which enhances the functional compensation capacity and stability of the rhizosphere microbial system in a high-salt environment [88]. Under heat stress, the rhizosphere microbial network is simplified. Edge number, average connectivity, and density decline, while path length increases and network stability decreases. By contrast, when heat stress occurs together with elevated CO_2_, the network becomes more complex, with increased edges and modularity that enhance both complexity and stability [89]. Heavy metal stress has been associated with reduced bacterial diversity in rhizosphere soils. Compared with bacteria, fungal communities often show stability under cadmium exposure and related stress conditions [90].

Different abiotic stresses are associated with shifts in rhizosphere microbial community structure and interaction patterns. These changes may affect the function and stability of the rhizosphere environment, with potential impacts on tea plant growth and quality-related metabolism.

## 4. Root Exudate–Microbiome Interactions Regulating Tea Quality Under Abiotic Stress

**Figure 1:**
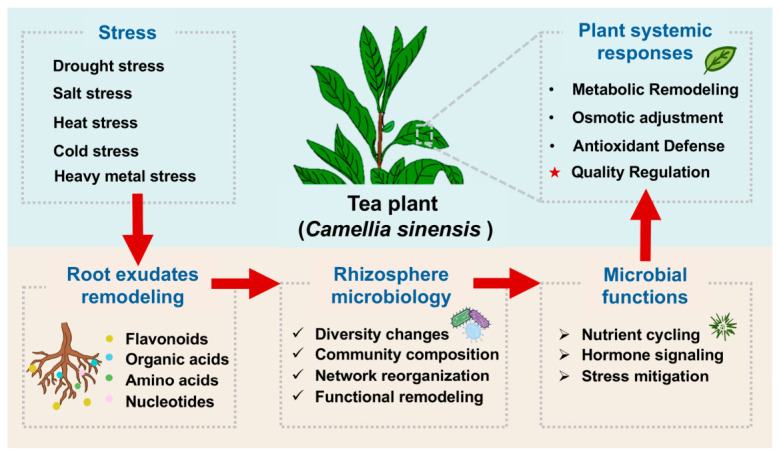
Root exudate–microbiome interactions regulating tea quality under abiotic stress. Tea plants subjected to abiotic stresses (drought, heat, salt, heavy metal, low temperature) reshape root exudate composition and selectively assemble the rhizosphere microbial community. These microbial changes, including diversity shifts, network reorganization, and functional remodeling, feedback to plant systemic responses such as metabolic remodeling, osmotic adjustment, antioxidant defense, and quality regulation. Through this cycle, the rhizosphere microbiome contributes to tea plant stress adaptation and tea quality formation.

Abiotic stress influences both root exudation and rhizosphere microbial communities in tea plants. These changes may subsequently affect nutrient metabolism and plant physiological processes. Root exudates and rhizosphere microorganisms jointly influence the accumulation of tea quality-related compounds, including amino acids, catechins, caffeine, polyphenols, and aroma compounds. These interactions ultimately contribute to the formation of an integrated feedback system linking tea plants, rhizosphere, and tea quality under abiotic stress (Figure 1).

### 4.1. Root Exudate-Mediated Microbial Recruitment

Under abiotic stress, plants change both composition and metabolite profile of their root exudates, including organic acids, amino acids and nucleotides. These changes then extend to the rhizosphere microbial community [18]. The specific exudates released depend on the type of stress. Under drought, plants release more organic acids such as lactic acid, citric acid, and succinic acid, and these compounds help recruit plant growth-promoting rhizobacteria to the root surface [13]. Tea plants under temperature stress secrete more amino acid, including L-theanine, asparagine, glycine, and γ-aminobutyric acid (GABA). These compounds reflect internal changes in carbon and nitrogen metabolism, while also functioning as nutrients and signaling molecules for beneficial rhizosphere microbes [4,11]. In tea plants exposed to salinity, the exudate profile changes toward nucleotides and purine derivatives. These compounds help recruit chemotactic microbial taxa including *Pseudomonas* to the root [71,91].

Tea plants also release species-specific secondary metabolites that act on rhizosphere microbial communities [92]. However, under different abiotic stresses, the specificity of these exudate signals in shaping the microbial assembly remains unclear. It is also not well understood whether these compounds recruit functionally distinct microbial assemblages or lead to partially redundant microbial functions. Previous studies have reported that flavonoids, coumarins, terpenes, alkaloids, and indole-related compounds contribute to nutrient utilization, plant defense, and microbe–plant interactions [93,94,95]. In tea plants, pathogen infection has been associated with changes in root exudate composition, including increased secretion of phenolic acids and flavonoids. These compounds may facilitate the enrichment of beneficial microorganisms such as *Pseudomonas*, *Bacillus*, and *Trichoderma*, which are often linked to enhanced plant resistance responses [96]. Overall, stress-induced changes in root exudates may alter rhizosphere microbial composition and function, with potential implications for tea plant growth and quality formation.

### 4.2. Microbial Regulation of Tea Quality Compounds

Rhizosphere microorganisms shape the biosynthesis of multiple compounds that determine tea quality, among them theanine and other free amino acids, polyphenols, catechins, and aroma volatiles. Tea cultivars with distinct rhizosphere bacterial communities also differ in the quality compounds. Cultivars with higher bacterial richness were reported to accumulate more polyphenols, theanine, and caffeine in their leaves [97].

Tea plants preferentially take up ammonium as their nitrogen source. Under abiotic stress, the ammonium available to roots is determined by the surrounding microbial community. Root-associated bacteria, such as *Bacillus* and *Pseudomonas*, can enhance ammonium uptake in tea plants, which is associated with higher theanine accumulation in leaves [17]. This effect likely involves coordinated regulation of ammonium transport, nitrogen assimilation and the GS-GOGAT pathway. However, whether these effects are maintained under abiotic stress has not yet been tested. Arbuscular mycorrhizal fungi (AMF) act through a different mechanism; it can enhance host nitrogen assimilation and support amino acid accumulation in the leaves under drought [98]. Theanine released from tea root can then reshape rhizosphere microbial communities, with feedback between tea plants and their microbiome [11]. In addition, rhizosphere microbes also act on catechins, polyphenols, and aroma compounds. The AMF genera *Paraglomus* and *Glomus*, both abundant in tea root, correlate positively with the catechin quality of green tea [99]. Inoculation studies have confirmed this effect, with AMF-treated plants accumulating more polyphenols and catechins under both drought and well-watered conditions [98]. Among bacterial groups, *Pseudomonas* and *Bacillus* contribute to quality compounds, affecting amino acid accumulation and the volatile profile of tea shoots [100]. Overall, rhizosphere microorganisms appear to participate in multiple metabolic pathways related to tea quality formation.

Shifts in rhizosphere microbial communities are often associated with changes in tea quality-related metabolites, including theanine, catechins, polyphenols, and volatile compounds. However, most available studies are based on correlative evidence, and direct causal relationships between stress-induced root exudates, microbial community dynamics, and the regulation of tea quality-related biosynthetic pathways have not been clearly established. Therefore, identifying the microbial taxa that contribute to these processes remains an important topic for future research.

## 5. Application Strategies for Microbial Regulation in Tea Plantations

**Figure 2:**
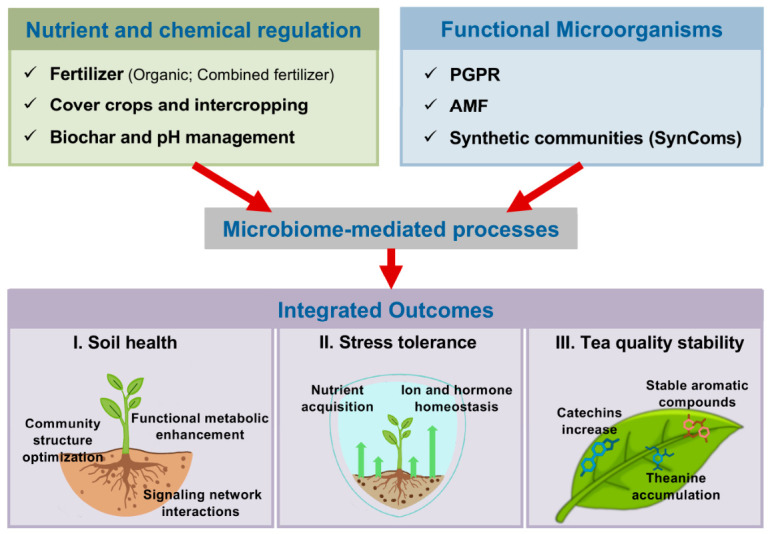
Microbiome-based management and integrated rhizosphere–plant system responses in tea plantations under abiotic stress. Nutrient and chemical regulation, and functional microorganisms jointly improve soil health, stress tolerance, and tea quality stability.

Recent studies on tea plant–rhizosphere interactions have provided new strategies for tea plantation management under abiotic stress. Current approaches mainly focus on optimizing rhizosphere nutrient conditions and applying beneficial microorganisms to improve stress tolerance and maintain tea quality (Figure 2).

### 5.1. Nutrient and Chemical Regulation of the Rhizosphere

Optimal application of chemical fertilizers can alleviate the inhibitory effects of drought, salinity, and other stresses on photosynthesis and growth by stabilizing plant carbon and nitrogen metabolism and improving osmotic regulation and carbohydrate metabolism [101,102]. Soil fertilization and chemical management directly influence the tea rhizosphere environment and the associated microbiome. The choice between chemical, organic, and combined fertilization strategies can shift the abundance and composition of beneficial microbes [103], with downstream effects on tea quality compounds including polyphenols, catechins, and amino acids [104]. Under abiotic stress, organic and combined fertilization can buffer the negative impacts of drought, salinity, and nutrient imbalance by maintaining microbial diversity, enhancing soil organic matter, and stabilizing nitrogen and carbon supply to tea plants.

Cover crops and intercropping are important agricultural practices for improving the rhizosphere microenvironment under abiotic stress. Under abiotic stress conditions, cover crops can influence rhizosphere microbial communities through the continuous input of organic carbon into the soil [105]. These microbial changes have been associated with improvements in soil structure, water-holding capacity, and nutrient availability, which may contribute to greater stress tolerance in crops [106,107]. In tea plantations, legume intercropping systems provide additional carbon and nitrogen inputs to the rhizosphere and are often accompanied by increases in plant growth-promoting bacteria and microbial nutrient turnover under stress conditions [108,109]. Green manure intercropping has also been reported to increase microbial functional diversity and improve the resistance of tea soils to drying–rewetting disturbances that mimic drought stress [110]. Field studies further suggest that ground cover management may affect plant responses to abiotic stress through changes in root growth, rhizosphere metabolites, and microbial community composition [111,112].

In addition to fertilization and cover cropping, regulation of the rhizosphere chemical environment may also help alleviate abiotic stress in tea plantations. Tea is grown on acidic soils with pH typically in the 4.5–5.5 range, and long-term cultivation tends to lower soil pH further, intensifying heavy metal accumulation. Biochar application is one of the more widely tested interventions for these problems. Previous studies suggest that biochar raises soil pH, lowers exchangeable aluminum, and increases cation exchange capacity, producing soil conditions more favorable to tea growth [113]. Biochar amendments have also been linked to changes in rhizosphere microbial communities, including increased abundance of beneficial bacterial and fungal groups [113,114]. Fertilization, intercropping, and biochar amendment share a common mechanism. Each improves soil chemistry and supports a more stable microbial community, thereby reducing stress pressure on tea plants.

### 5.2. Functional Microorganisms for Stress and Quality Regulation

#### 5.2.1. Plant Growth-Promoting Rhizobacteria

Under abiotic stress, inoculation with plant growth-promoting rhizobacteria (PGPR) is recognized as an effective microbial intervention strategy. PGPR improve the stress adaptability of tea plants and contribute to quality formation by enhancing nutrient acquisition, promoting plant growth, and improving tolerance to abiotic stresses [115,116]. Tea-specific PGPR have been isolated from acidic tea rhizosphere soils. These rhizobacteria include several stress-tolerant genera, such as *Bacillus*, *Pseudomonas*, *Burkholderia*, and *Paenibacillus*. They can tolerate drought, salinity, and low pH, while also promoting tea seedling growth under different soil conditions [117].

Application of PGPR in tea plantations can improve stress tolerance and tea quality under abiotic stress. PGPR inoculation reshapes the rhizosphere microbial community structure of tea plants. It increases microbial diversity and richness and enriches stress-tolerant and metabolically active groups. These changes improve soil nutrient status and enhance microbial functional activity [118,119]. Application of Proteobacteria PGPR through root irrigation can positively affect tea plant growth and chlorophyll accumulation. Changes in soil nutrient status and rhizosphere microbial communities have also been observed following PGPR treatment [120]. In field conditions, *Burkholderia* and *Pseudomonas* inoculation has been associated with increased tea yield and changes in tea quality-related metabolites. For example, higher theanine accumulation and lower polyphenol and caffeine contents have been observed after PGPR treatment [121]. These findings indicate the potential value of PGPR in stress management for tea cultivation.

#### 5.2.2. Arbuscular Mycorrhizal Fungi

Arbuscular mycorrhizal fungi (AMF) may increase the efficiency of nutrient absorption and transport in plant roots. Previous studies have shown that AMF can contribute to plant adaptation under drought, salt, and heavy metal stress conditions [98,122,123].

In tea plants, AMF colonization has been linked to changes in leaf metabolite profiles, including amino acids, soluble sugars, polyphenols, and flavonoids [124,125]. Under drought conditions, AMF inoculation may promote nitrogen assimilation and amino acid accumulation in tea leaves and roots, accompanied by increased polyphenol and catechin contents [98]. Studies under salt stress have further shown that AMF can influence osmotic adjustment and antioxidant responses in tea plants, while reducing excessive lignin and cellulose accumulation that may negatively affect leaf tenderness [122]. AMF have also been reported to alleviate heavy metal stress, including manganese toxicity in acidic tea soils [123]. These effects are associated with changes in antioxidant activity and quality-related metabolism. In addition, AMF inoculation has been linked to accelerated tea bud growth and altered metabolite composition, with higher theanine levels and lower polyphenol and caffeine contents in green tea [126].

PGPR and AMF can influence plant stress responses and secondary metabolism through phytohormones, volatile compounds, antimicrobial metabolites, and other microbial signals. These signals may regulate stress-responsive transcriptional networks in tea plants and affect transcription factors and biosynthetic pathways involved in nitrogen assimilation, phenylpropanoid metabolism, purine alkaloid metabolism, and aroma volatile formation. However, the transcription factors and gene clusters directly linking microbial signals to tea quality compound accumulation remain unclear. Addressing this question will require integrated multi-omics analyses and functional validation using microbial consortia or synthetic communities.

#### 5.2.3. Multifunctional Microbial Consortia and Synthetic Communities

Single function microbial inoculants rarely establish stably in tea plantation soils, and their functional benefits tend not to persist over time. Therefore, recent work has moved toward multifunctional formulations and synthetic communities (SynComs) [127,128]. By bringing together microbes with complementary ecological roles, SynComs tend to form more stable rhizosphere assemblies than single-strain inoculants. Previous studies have shown that SynComs isolated from plants native to extreme habitats, which can colonize non-sterile soils, modulate stress-responsive gene expression in the host, and help maintain ion homeostasis under environmental stress [129]. In tea plants, a SynCom from the rhizosphere of the high-theanine cultivar Rougui enhances ammonium uptake and raises leaf theanine content [17].

The stability and function of SynComs depend on the interactions between their members. Their growth-promoting and stress-mitigating effects do not come solely from plant physiological adjustments; resource partitioning and functional complementarity among rhizosphere members also play a substantial part. For example, under heavy metal stress, the core strains of SynComs can metabolize root exudates sequentially rather than simultaneously. Thereby, interspecific competition is reduced, and carbon utilization in the rhizosphere becomes more efficient. The cooperative relationship enhances community colonization capacity and promotes plant growth [130]. Under salt stress, salt-tolerant SynComs improve plant adaptation by reducing oxidative damage, regulating osmotic substances, and maintaining ion homeostasis [131]. Under aluminum stress, synthetic microbial communities promote lateral root development, thereby improving tea plant tolerance to aluminum toxicity [16]. These findings suggest that SynComs deliver reliable growth-promoting and stress-mitigating benefits across abiotic stresses, with cooperation among member strains as the underlying mechanism.

## 6. Conclusions and Future Perspectives

This review summarizes how abiotic stress reshapes the rhizosphere microbial community of tea plants, and how those communities influence tea quality. Under stress, tea plants change the composition of their root exudates, and the exudates selectively recruit specific microbial groups. The recruited microbes drive nitrogen and carbon cycling, relay hormone signals, and contribute to the biosynthesis of metabolites, ultimately shaping the accumulation of tea quality-related compounds. Accordingly, organic amendments, intercropping systems, and microbial inoculation have been investigated as managements to enhance stress tolerance and stabilize tea quality. Despite this progress, several questions remain unresolved. Future studies should address the following directions:

(1) Mechanistic dissection of the “stress–exudate–microbiome-quality” chain. Direct evidence linking stress-induced changes in root exudates to specific tea quality compounds via microbial mediation is still limited. Identifying key microbial taxa will require validation using simplified SynComs, microbial add-back/removal experiments, and gnotobiotic or semi-controlled tea cultivation systems. Future research should distinguish microbial taxa that are merely associated with tea quality traits from those that are sufficient and necessary in regulating nitrogen uptake, hormone signaling, and the accumulation of tea quality-related compounds under abiotic stress.
(2) Microbial regulation of specific tea quality compounds. Identifying which microbial groups most strongly influence which key compounds could enable compound-targeted microbial interventions. The most commercially relevant quality compounds include theanine, catechins, and specific aroma volatiles. Since some key compounds are also released as root exudates, future studies combining exudate metabolomics, microbiome profiling, and SynCom validation may help determine whether they recruit functionally distinct or redundant microbial assemblages under abiotic stress.
(3) Integration of spatial, single-cell, and systemic multi-omics. Current studies mainly combine rhizosphere metagenomics with plant metabolomics, providing limited information at the cellular level. Incorporating single-cell/nucleus RNA sequencing and spatial transcriptomics into multi-omics studies may improve our understanding of how tea root cells respond to microbiome-derived signals and abiotic stress [132].
(4) Field-ready synthetic communities. Tea-specific SynComs have been validated mainly in laboratory or greenhouse experiments. An important next step is to design SynComs that colonize stably and retain their function under field conditions, particularly combining PGPR, AMF, and others beneficial microbes.
(5) Climate-resilient tea plantation microbiomes. An open question is how tea rhizosphere communities respond to co-occurring climate stresses, and whether microbial interventions can buffer these effects across multiple growing seasons.

Despite these advances, most research into the tea rhizosphere microbiome still stops at community profiling. Rhizosphere microbiome research needs to move from descriptive surveys toward causal evidence and field validation. This progress is the basis for using rhizosphere microbes in climate-resilient tea plant cultivation.

## Figures and Tables

**Table 1:** Changes in key microbial taxa in the rhizosphere under different abiotic stresses.

Stress Type	Plant/System	Enriched Microbial Communities	Reduced Microbial Communities	References
Drought stress	*Oryza sativa*/rhizosphere	(Phylum) Actinobacteria (Genus) *Streptomyces*, *Actinoplanes*, *Catellatospora*, *Dactylosporangium*, *Pseudonocardia*	(Phylum) Acidobacteria	[64]
	*Trifolium subterraneum*/rhizosphere	(Phylum) Actinobacteria, Firmicutes	(Phylum) Proteobacteria, Verrucomicrobia, Gemmatimonadetes	[65]
	*Arachis hypogaea*/rhizosphere	(Phylum) Cyanobacteria, Planctomycetes (Genus) *Sphingomonas*, *Streptomyces*,	(Phylum) Proteobacteria, Chloroflexi, Verrucomicrobia (Class) *Gammaproteobacteria*, *Deltaproteobacteria* (Genus) *Chthoniobacter*, *norank_c_KD4-96*	[55]
	*Lasiurus sindicus*/rhizosphere	(Phylum) Actinobacteria, Firmicutes, (Genus) *Bacillus*, *Actinomycetes*	(Phylum) Proteobacteria	[66]
	Spring wheat/rhizosphere	(Genus) *Pseudomonas*, *Streptomyces*	Not specified *	[67]
	*Zea mays*/rhizosphere	(Family) Aspergillaceae	(Family) Sphingomonadaceae, Sphingobacteriaceae, Sarocladiaceae	[68]
Salt stress	*Matricaria chamomilla*/rhizosphere	(Family) Geminicoccaceae, Rokubacteriaceae, Comamonadaceae, Gemmatimonadaceae (Genus) *Vagum*, *Nemalophilum*, *Funneliformis*, *Candida*	(Phylum) Chytridiomycota (Family) Xanthobacteraceae (Genus) Pedomicrobium, Bauldia, Acidibacter, Hyphomicrobium, Simmonsii	[57]
	*Oryza sativa*/rhizosphere	(Phylum) Bacteroidota, Cyanobacteria	(Phylum) Firmicutes, Acidobacteriota, Myxococcota	[63]
	*Arachis hypogaea*/rhizosphere	(Phylum) Cyanobacteria, Proteobacteria (Genus) *Rhizobium*	(Phylum) Acidobacteria	[69]
	Maize fields/soil	(Phylum) Firmicutes, Bacteroidetes, Mortierellomycota	(Phylum) Chloroflexi, Acidobacteria, Rokubacteria, Nitrospirae	[70]
	*Leymus chinensis*/rhizosphere	(Phylum) Myxococcota, Bacteroidota	Not specified *	[71]
Heat stress	Paddy field/rhizosphere	(Phylum) Firmicutes (Genus) *Alicyclobacillus*, *Bacillus*, *Oxalophagus*	(Phylum) Actinobacteria, Chloroflexi (Genus) *Oryzihumus*, *Catenulispora*, *Kitasatospora*	[61]
	*Bamboo*/soil	(Phylum) Proteobacteria, Actinobacteria, Bacteroidetes (Genus) *Bradyrhizobium*, *Burkholderia*, *Mucilaginibacter*	(Phylum) Acidobacteria, α-Proteobacteria	[72]
	Agricultural soil/soil	(Phylum) Firmicutes, Proteobacteria (α-, β-, γ-) (Genus) *Sporosarcina*, *Paenisporosarcina*, *Rhizomicrobium*, *Burkholderia*	Not specified *	[73]
	*Triticum aestivum*/soil	(Phylum) Actinobacteria, Bacteroidetes, TM7	(Phylum) Proteobacteria, Acidobacteria, Chloroflexi, Firmicutes, Nitrospirae, Verrucomicrobia	[74]
	Tropical soil/soil	(Phylum) Actinobacteria, Chloroflexi (Genus) *Gaiella*, *Nocardioides*	(Phylum) Proteobacteria, Acidobacteria, Planctomycetes (Genus) *Bradyrhizobium*, *Mycobacterium*, *Tepidisphaera*, *Paludibaculum*	[75]
Cold stress	*Zea mays*/rhizosphere	(Family) Comamonadaceae, Pseudomonadaceae	(Family) Streptomycetaceae	[76]
	*Tetrastigma hemsleyanum* Diels & Gilg/rhizosphere	(Phylum) Bacteroidetes, Chloroflexi (Genus) *Chryseolinea*	Not specified *	[77]
	*Oryza sativa*/rhizosphere	(Phylum) Chloroflexi, Acidobacteriota	Not specified *	[78]
	*Lolium perenne*/rhizosphere	(Phylum) Firmicutes, Acidobacteriota, Actinobacteriota (Genus) *Pseudomonas*	(Phylum) Proteobacteria	[79]
Heavy metal stress	*Camellia sinensis*/rhizosphere	(Genus) *Bacillus*, *Alicyclobacillus*, *Nitrospira*, *Sporosarcina*, *Gaiella*, *Tumebacillus*, *Paenibacillus* *Trichoderma*, *Talaromyces*, *Fusarium*, *Metarhizium*, *Aspergillus*, *Rhodotorula*	(Genus) *Thermosporothrix*, *WPS_2_genera_incertae_sedis*, *Coniosporium*, *Exophiala*, *Hamigera*	[58]
	*Oryza sativa*/rhizosphere	(Phylum) Proteobacteria, Bacteroidetes, Firmicutes (Genus) *Pseudomonas*, *Sulfuricurvum*, *Bellilinea*	(Phylum) Acidobacteria, Chloroflexi (Genus) *Longilinea*	[80]
	*Vaccinium corymbosum*/rhizosphere	(Class) Eurotiomycetes, Sordariomycetes (Genus) *Coniochaeta*, *Talaromyces*	(Class) Agaricomycetes, Archaeorhizomycetes	[81]
	*Populus*/rhizosphere	(Phylum) Chloroflexi, Rhodanobacter, Gemmatimonadetes (Genus) *Bradyrhizobium*, *Streptomyces*, *Knoellia*, *Luedemannella*, *Sphingomonas*	(Phylum) Nitrospirae, Acidobacteria (Genus) *Gaiella*	[82]
	*Camellia sinensis*/rhizosphere	(Genus) *ADurb.Bin063_1*, *Puia_dinghuensis*, *Acidobacteriaceae_bacterium_K5*, *Bradyrhizobium*, *Edaphobacter*	(Genus) *Acidipila silvibacterium*, *Occallatibacter*	[83]

* Not specified: the original study did not report the reduced microbial communities.

## Data Availability

This is a review article, and no new data were generated or analyzed in this study. All data cited are from publicly available sources as referenced in the manuscript.

## Abbreviations

The following abbreviations are used in this manuscript:

AMF	Arbuscular mycorrhizal fungi
GABA	γ-aminobutyric acid
PGPR	Plant growth-promoting rhizobacteria
ROS	Reactive oxygen species
SynComs	Synthetic communities
VOCs	Volatile organic compounds
